# Football Was the Most Common Sport among 344 Consecutive Athletes Treated Surgically for Jumper's Knee at an International Tendon Clinic

**DOI:** 10.1155/2024/5534733

**Published:** 2024-06-29

**Authors:** Håkan Alfredson, Christoph Spang, Markus Waldén

**Affiliations:** ^1^ Sports Medicine Unit Department of Community Medicine and Rehabilitation Umeå University, Umeå, Sweden; ^2^ Capio Ortho Center Skåne, Malmö, Sweden; ^3^ Institute of Sports Science University of Würzburg, Würzburg, Germany; ^4^ Private Orthopaedic Spine Center, Würzburg, Germany; ^5^ Unit of Public Health Department of Health Medicine and Caring Sciences Linköping University, Linköping, Sweden

## Abstract

**Objectives:**

Jumper's knee, or proximal patellar tendinopathy, is commonly seen among athletes in leg explosive sports, and for a subgroup surgical treatment is needed. The aim of this study was to identify what type of sports were most frequent among athletes treated surgically for Jumper's knee at an international tendon clinic during a consecutive 13-year period.

**Methods:**

The study included 344 consecutive patients (306 males, mean age 27 years, range 17–58; 38 females, mean age 24 years, range 18–44) from 21 different countries seeking help for therapy-resistant jumper's knee. There were 274 elite athletes, 168 being full-time professionals. All were diagnosed to have tendinopathy in the proximal patellar tendon and were operated on with ultrasound- and Doppler-guided arthroscopic shaving surgery.

**Results:**

The single most common sport was football (*n* = 95, 28%), followed by rugby (*n* = 37, 11%) and handball (*n* = 32, 9%), with 117 (34%) playing at a professional level. The rest of the athletes participated in 17 other different elite sports and nine recreational sports (running/jogging, padel, squash, biking, gym training, bowling, cheerleading, dancing, and ultimate frisbee).

**Conclusions:**

Football was the most common sport among patients requiring surgical treatment for jumper's knee, constituting 28% of all patients, and together with rugby and handball they constituted almost half of all patients. There was a wide sport distribution with 29 different team and individual sports represented.

## 1. Introduction

Jumping sports like volleyball and basketball, with repetitive high and explosive loads on the knee extensor mechanism, are known to be associated with Jumper's knee, or proximal patellar tendinopathy, but several other sports, both on elite and non-elite levels, are also represented [1, 2]. The condition can be difficult to treat, but different types of loading regimens are often successful [3, 4]. For a subgroup of patients, however, non-surgical treatment gives insufficient symptom relief and surgery might be needed [5–7]. It is tempting to believe that the most advanced tendon and bony changes are the ones that require surgical treatment. Therefore, it is interesting to know whether athletes in certain sports are more frequently represented among surgically treated patients, but this is essentially unclear from the current literature [8].

This study aimed to identify what type of sports was most frequent among athletes treated surgically for Jumper's knee at an international tendon clinic during a consecutive 13-year period.

## 2. Materials and Methods

In this study, consecutive patients that were operated for therapy-resistant Jumper's knee at an international tendon clinic in Sweden, between 2011 and 2023, were included. All patients had long-standing pain (>6 months) and were not able to train and/or compete at their desired level in their sport. A wide range of non-surgical treatment methods had been tried without success. Frequencies of sport types for each patient were determined.

**Figure 1:**
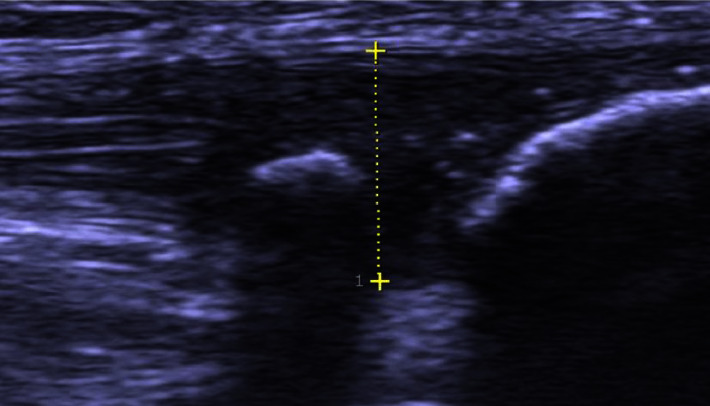
Grey scale ultrasound examination-longitudinal view on a patient with proximal patellar tendinopathy. There is a thickening of the proximal patellar tendon (marked) including tendinopathy on the dorsal side of the tendon, a loose intratendinous bone fragment and an indication of a possibly sharp bone edge in the patellar tip.

All patients were examined clinically by one or two experienced orthopaedic surgeons. Immediately following the clinical examination patients were also examined bedside with high-resolution grey scale ultrasound (US) and colour Doppler (CD) using a linear multifrequency (8–13 MHz) probe (S-500, Siemens AG, Germany), confirming structural changes typical for patellar tendinopathy (Figure 1). The US + CD-guided wide awake local anaesthetic no tourniquet (WALANT) arthroscopic shaving procedure [7] was used for the surgical treatment and was performed exclusively by one or two orthopaedic surgeons.

### 2.1. Ethical Approval

Medical Faculty of the Karolinska Institute, Stockholm (Dnr: 2011-929-32).

### 2.2. Statistical Analyses

Data were stored in a password-protected Excel file. Descriptive statistics with absolute numbers and percentages are reported. No group-wise comparisons were made.

## 3. Results

This study included 344 consecutive patients from 21 different countries. There were 306 males (mean age 27 years, range 17–58) and 38 females (mean age 24 years, range 18–44). Altogether 274 patients were elite athletes, 168 being full-time professionals, and 70 were active on a non-elite amateur or recreational level.

As outlined in Table 1, the single most common sport was football (*n* = 95, 28%), followed by rugby (*n* = 37, 11%) and handball (*n* = 32, 9%), 117 of these playing at a professional level (football *n* = 58, rugby *n* = 35, and handball *n* = 24), 8 playing at a non-professional elite level (handball *n* = 8) and 39 at a non-elite amateur level (football *n* = 37 and handball *n* = 2). The rest of the elite athletes participated in 17 other different sports.

Sports with only male patients were Gaelic football, ice hockey, basketball, powerlifting CrossFit, triathlon/ultramarathon, Australian rules football, martial arts, cricket, American football, floorball, hurling, and cross-country skiing.

Additional sports with patients active on a recreational level were gym training (*n* = 32, 3 female), running/jogging (*n* = 28, 2 female), biking (*n* = 2), bowling (*n* = 1), cheerleading (*n* = 1, 1 female), dancing (*n* = 1), padel (*n* = 2), squash (*n* = 1), and ultimate frisbee (*n* = 2).

## 4. Discussion

The current study showed that football was the most common sport among patients requiring surgical treatment for Jumper's knee during a 13-year period at an international tendon clinic.

It could be argued that this mainly reflects the situation that football is the most popular sport both in Sweden and in the world, but the sample included a large number of international patients from 21 different countries, with Australia and New Zealand being most far away. The sample thereby included athletes being active in many different sports, reflecting various popularities in different countries. Even if football, rugby, and handball players constituted almost half of all patients, a wide range of sports were represented, showing that Jumper's knee not only affects high-level athletes in classic jumping sports.

It has previously been shown that Jumper's knee affected 2.4% of professional/elite male football players in Europe and constituted 1.5% of all time-loss injuries in this setting [9]. Even if many of these injuries were recurrent, it is not known from that study how many injuries underwent surgery, and we are not aware of any other study reporting on the surgery need for Jumper's knee among football players. Importantly, it has been shown that the number of sprints and other high-intensity actions in men's professional football have increased during the 2000s [10], and it can be speculated if these demanding actions can trigger the development of Jumper's knee among football players. An interesting, and somewhat worrying, observation was that 23 of the 95 football players (24%) in the current study were younger than 20 years, many being so-called elite academy players. This could potentially indicate that there is a tendon overload already in young ages, creating symptomatic tendon changes that are so serious that surgical treatment is needed.

Previous studies suggested that Jumper's knee is much more frequently reported and diagnosed in male athletes compared to female athletes [1, 2]. In a recent study, jumper's knee was equally distributed in males and females [11]. In the present study, there was a strong male dominance for all sports in this study except for handball, where more than one-third of the players were females. Interestingly, classic jumping sports such as basketball and volleyball were less frequently represented in this sample (only ten basketball players and seven volleyball players). The most plausible explanation here is that our sample represents surgically treated patients only, and maybe the tendon changes and symptoms related to Jumper's knee in volleyball and basketball are on a level where they can manage non-surgically without time loss from sport or reduced performance. These thoughts are supported by the findings by Tayfur and colleagues showing that volleyball, handball, and basketball players with Jumper's knee are able to play more than others [11]. This raises the important question if different sports induce different types of tendinopathic and bony changes in the proximal patellar tendon insertion. Also, is there a more serious variant where surgery might be needed because there is pain on a level where the athlete cannot train and play at the desired level and performance (such as in football, rugby, and handball)? The findings in this study, and these questions, warrant further sport-specific research in this field.

This study has a number of limitations. First, the total number of athletes-at-risk and the overall exposure hours are unknown so our study cannot compare the sports according to prevalence or incidence. Second, patients were referred to us if there was longstanding pain for more than half a year despite various non-surgical treatment modalities, but we do not know how many patients who had similar symptoms chose to end their careers or continued with their sport, but with intermittent time loss and reduced performance. Third, the sample consists of patients deciding to have surgery at this particular tendon clinic where the method used is the minimally invasive US + CD arthroscopic shaving procedure, but there are likely patients operated elsewhere with other surgical methods.

## 5. Conclusions

In conclusion, in this consecutive surgical case series football players were the most common patients requiring surgical treatment for Jumper's knee, constituting 28% of all patients. Altogether, 29 different sports were represented which illustrates that many other activities and actions than those described in classic jumping sports are associated with Jumper's knee.

## Figures and Tables

**Table 1:** Sport representation among professional/elite level athletes treated surgically for Jumper's knee.

Sport	No. of athletes (%)
Football	95 (28%) (i) 58 professional, 2 females (ii) 37 non-elite, 7 females

Rugby	37 (11%) (i) 35 professional, 1 female (ii) 2 non-elite, 1 female

Handball	32 (9%) (i) 12 females

Gaelic football	12 (3%)

Track and field	12 (3%) (i) 2 females

Alpine skiing including freeskiing	11 (3%) (i) 4 females

Ice hockey	11 (3%)

Basketball	10 (3%)

Powerlifting	8 (2%)

CrossFit	7 (2%)

Volleyball	7 (2%) (i) 1 female

Triathlon/ultramarathon	6 (2%)

Australian rules football	5 (1%)

Cricket	5 (1%)

Martial arts	5 (1%)

American football	4 (1%)

Floorball	3 (<1%)

Tennis	2 (<1%) (i) 2 females

Cross-country skiing	1 (<1%)

Hurling	1 (<1%)

Total	274 (100)

Sports listed according to frequency. All athletes were professional/elite and male except for where specified.

## Data Availability

The data used to support the findings of this study are available from the corresponding author upon reasonable request.
